# Melatonin Attenuates Oxidative Damage Induced by Acrylamide In Vitro and In Vivo

**DOI:** 10.1155/2015/703709

**Published:** 2015-06-21

**Authors:** Xiaoqi Pan, Lanlan Zhu, Huiping Lu, Dun Wang, Qing Lu, Hong Yan

**Affiliations:** ^1^Department of Health Toxicology, MOE Key Lab of Environment and Health, School of Public Health, Tongji Medical College, Huazhong University of Science and Technology, Wuhan 430030, China; ^2^Institute for Environmental Medicine, MOE Key Lab of Environment and Health, School of Public Health, Tongji Medical College, Huazhong University of Science and Technology, Wuhan 430030, China; ^3^Sanya Center for Disease Control and Prevention, Hainan 572000, China; ^4^Shanghai Songjiang District Center for Disease Control and Prevention, Shanghai 200000, China

## Abstract

Acrylamide (ACR) has been classified as a neurotoxic agent in animals and humans. Melatonin (MT) has been shown to be potentially effective in preventing oxidative stress related neurodegenerative disorders. In this study, whether MT exerted a protective effect against ACR-induced oxidative damage was investigated. Results in cells showed that reactive oxygen species (ROS) and malondialdehyde (MDA) significantly increased after ACR treatment for 24 h. MT preconditioning or cotreatment with ACR reduced ROS and MDA products, whereas the inhibitory effect of MT on oxidant generation was attenuated by blocking the MT receptor. Increased DNA fragmentation caused by ACR was significantly decreased by MT coadministration. In vivo, rats at 40 mg/kg/day ACR by gavage for 12 days showed weight loss and gait abnormality, Purkinje cell nuclear condensation, and DNA damage in rat cerebellum. MT (i.p) cotreatment with ACR not only recovered weight and gait of rats, but also decreased nuclear condensation and DNA damage in rat cerebellum. Using MDA generation, glutathione (GSH) level, superoxide dismutase (SOD), and glutathione peroxidase (GSH-Px) activities in rat cerebellum as indicators, MT alleviated ACR-induced lipid peroxidation and depressed antioxidant capacity. Our results suggest that MT effectively prevents oxidative damage induced by ACR.

## 1. Introduction

Acrylamide (ACR) has been widely used in various industrial settings, such as wastewater management, cosmetic manufacturing, dye synthesis, and scientific laboratories for the electrophoretic separation of macromolecules. ACR is found to be present in starchy food under high temperature [1, 2]. As a well-documented neurotoxic agent in both humans and laboratory animals, its existence is a worldwide concern [3]. Subchronic, low-level occupational human exposure to ACR can lead to neurotoxicity characterized by ataxia, skeletal muscle weakness, and numbness of the hands and feet. Many studies have indicated that rats treated orally with ACR at 20~50 mg/kg/day can produce cumulative neurotoxicity, primarily exhibiting gait abnormality [4–6]. As is well known, the cerebral cortex is one of the advanced areas in the nervous system, and it controls sensory and movement in humans and animals, whereas the cerebellum maintains the balance and coordination of the body. However, the exact mechanism underlying ACR neurotoxicity remains unclear.

Oxidative damage has been suggested to be one of the mechanisms of neurotoxicity induced by ACR [7]. An imbalance between the overproduction of reactive oxygen species (ROS) and elimination of free radicals induces oxidative damage. Excesses in free radical generation induced by oxidative metabolism imbalance result in a series of changes, including protein and DNA injury, energy deficiency, inflammation, and mitochondrial dysfunction. Some studies have suggested that ACR can lead to an imbalance in nervous tissue and the sciatic nerve by enhancing lipid peroxidation and reducing antioxidant capacity, as evidenced by enhanced levels of ROS and MDA, accompanied with a decrease in the levels of superoxide scavenging enzymes SOD, catalase, and glutathione peroxidase (GSH-Px) [8–10].

Melatonin (MT), an efficient free radical scavenger, is the main hormone secreted by the pineal gland. It has been found to exert protective effects on a number of pathological damages including shock, ischemia reperfusion, and inflammation [11, 12]. In addition, MT is a potent free radical scavenger with a broad spectrum of antioxidant capacity [13–16]. It readily crosses various physiological barriers because of its high lipophilicity and hydrophilicity, such as the blood-brain barrier. Thus, its pharmacological use may potentially protect against oxidative damage existing anywhere in the organism. MT plays a crucial role in preventing oxidative damage of biological macromolecules caused by oxidative substance and protecting the vitality of antioxidant enzymes in the body [14]. Considering the proposed dosage of MT (1~3 mg) in prevention and based on previous studies, MT at doses of 5–10 mg/kg exhibited an effective cellular antioxidation capacity [17, 18]. The absolute bioavailability of MT ranges from 1% to 37% (mean = 8.6%~3.9% and 16.8%~12.7% for male and female subjects, resp.) [19].

In this study, ACR-poisoning models were established both in vitro and in vivo. To explore whether MT intervention can alleviate oxidative damage induced by ACR, oxidative and antioxidative parameters including ROS production, glutathione (GSH) consumption, and SOD and GSH-Px activities, as well as neurological impairment, morphological alteration, and DNA damage, were assayed. Moreover, luzindole (a novel MT receptor antagonist) was added to further study the possible mechanism of MT intervention in vitro. Luzindole acts as a selective MT receptor antagonist, with approximately 11~25-fold greater affinity for the MT receptors [20]. Conclusions of the paper will provide evidence for the chemoprevention of ACR toxicity.

## 2. Materials and Methods

### 2.1. Chemicals and Materials

Acrylamide (ACR), melatonin (MT), and other chemicals (all 99.9% purity) were obtained from Sigma Chemical Co. All solvents were of analytical grade and Milli-Q water was used throughout the analyses. Malonaldehyde (MDA), superoxide dismutase (SOD), glutathione (GSH), and glutathione peroxidase (GSH-Px) detection kits were provided by the Nanjing Jiancheng Bioengineering Institute (Nanjing, China). Fluorescent probe 2,7-dichlorofluorescein diacetate (DCFH-DA), Hoechst 33258 dye, normal-melting-point agarose (NMPA) gel, low-melting-point agarose (LMPA) gel, and ethidium bromide (EB) were from Sigma Co. RPMI-1640 culture medium and fetal bovine serum were purchased from GIBCO. 3-(4,5-Dimethylthiazol-2-yl)-2,5-diphenyltetrazolium (MTT) cell viability assay kit was from Promoter Biotechnology Inc. (Nanjing, China).

### 2.2. Cell Culture

Well-differentiated rat pheochromocytoma (PC12) cells induced by nerve growth factor were obtained from Shanghai Institutes for Biological Sciences, Chinese Academy of Cell Resource Center (Shanghai, China). Cells were maintained in RPMI-1640 with 10% heat-inactivated fetal bovine serum (FBS) (containing 100 U/mL penicillin and 100 U/mL streptomycin) and incubated at 37°C with 5% CO_2_. Culture medium was renewed every 2 days. Cells were collected and new culture bottles were seeded every 3 days. After incubation for 24 h, the cells were cultured with serum-free medium containing different concentrations (1.25, 2.5, and 5 mmol/L) of ACR dissolved in water for 1, 3, 6, 12, and 24 h. MT was dissolved in absolute ethanol. The cells in the vehicle control group were incubated with culture containing a final concentration of 0.1% ethanol, as well as in the experimental groups. Intervention with MT (50 *μ*mol/L) in cells was conducted at pre-24 h, simultaneously, and post-3 h with ACR (2.5 mmol/L) for the following experiments.

### 2.3. Cell Viability Assay

Cell viability was measured indirectly by MTT assay. PC12 cells were seeded in a 96-well plate at a density of 2 × 10^5^ cells/well in RPMI-1640 containing 10% FBS. The medium was replaced with the same medium containing ACR, or ACR with MT, whereas control cells were grown in RPMI-1640 containing equal water solvent. After incubation for 24 h, cells were treated with MTT solution for 4 h at 37°C. The solution was then removed, and formazan crystals were dissolved in dimethyl sulfoxide (DMSO) solutions. The absorbance at 490 nm was determined in a microplate reader. The results are expressed as the percentage of MTT reduction relative to the absorbance of control cells.

### 2.4. Animal Treatment

Healthy adult Sprague-Dawley male rats (6~7 weeks, 180~200 g) were obtained from the experimental animal center of Tongji Medical College, Huazhong University of Science and Technology in China. Individual weights were recorded, and detailed physical examinations were performed during the one-week adaptation period to ensure that animals were healthy. Each 3 rats were housed in a cage, experienced a 7-days adaptation period, and all rats were given free access to commercial laboratory feed and tap water during the nonexposure period. Rats were divided randomly into 4 groups (*n* = 9), namely, control group, ACR group, MT group, and MT + ACR cotreatment group.

ACR was dissolved in normal saline, whereas MT was dissolved in absolute ethanol and then diluted in saline to obtain a final alcohol concentration of 1% ethanol. MT (5 mg/kg/day, i.p) or vehicle (1% alcohol in saline) was administered 5 min prior to ACR (40 mg/kg b. w/day) oral administration for 12 days. The body weights were determined every 3 days. After 24 h following the last administration, rats were sacrificed for science. All organs were separated on ice and saved at 80°C for the next test. The study was performed in accordance with the guidelines for the care and use of laboratory animals, prepared by the National Institute of Health, USA (Guide for the Care and Use of Laboratory Animals, 1996).

### 2.5. Behavioral Index (Gait Scores) Examination in Rats

Gait scores were examined according to a previously described method [33]. On days 0, 3, 6, 9, and 12 of ACR with or without MT and luzindole cotreatment, rats were placed in the ground and allowed freedom of movement. Each one was observed for 3 min, and the gait score was assessed in terms of the following standards: 1: an unaffected or normal gait; 2: a slightly affected gait (hind limb weakness, slight ataxia, and foot splay); 3: a moderately affected gait (less active, foot splay with moderate limb spread during ambulation); and 4: a severely affected gait (foot splay, severe hind limb weakness, dragging hind limbs, and inability to rear).

### 2.6. Immunohistochemistry for Morphological Observation

Immunohistochemistry was used for morphological changes in rat brain tissues. The sagittal plane of rat cerebellum or cortex was fixed with 10% formaldehyde. The samples were dehydrated orderly in a series of ethanol concentrations (75%, 85%, 90%, and 95%) for 2 h each time and in absolute ethanol for 1.5 h. A total of 116 samples were soaked in xylene twice for 30 min each time to increase the transparency of samples. Thereafter, samples were placed into melted soft wax for 15 min. After specimens were embedded by a paraffin embedding apparatus, they were cut into slices at 37°C overnight. Slices were transferred into xylene for 15 min twice to remove paraffin. Subsequently, slices were rinsed by ethanol (100%, 95%, 90%, and 85%) in turn, stained by hematoxylin for 3 min, differentiated by hydrochloric acid-alcohol for 20 s, and stained by eosin for 40 s. Slices were dehydrated with ethanol (85%, 90%, 95%, and 100%) for 2 min. All slices were placed into xylene three times, for 2 min per time. Subsequently, slices were immobilized by gum and dried in an incubator. Each slice was randomly observed through 6 visions by an Olympus CH-20 optical microscope, and micrographs were obtained from Nikon-Coolpix 4500 digital camera.

### 2.7. DNA Condensation and Nuclear Fragmentation

Hoechst 33258 fluorescent dye was used to determine DNA condensation and nuclear fragmentation of apoptosis cells. PC12 cells were seeded in a six-well plate at a density of 2 × 10^5^ cells/well. After treatment with or without ACR for 24 h at 37°C, cells were washed with PBS and fixed with 4% paraformaldehyde for 20 min at 4°C. The fixed cells were washed with PBS and stained with 5 *μ*g/mL Hoechst 33258. After incubation for 15 min, the cells were washed with PBS, and photos were taken under an inverted fluorescence microscope (IX71, Olympus, Japan).

### 2.8. Single Cell Gel Electrophoresis Assay (Comet Assay)

DNA damage in the cerebellum and cortex of rats was detected by single cell gel electrophoresis (comet assay). The suitable tissues were prepared into a single cell suspension, mixed with moderate LMPA (37°C), and then transferred to slides. The slides were cooled by 95% alcohol overnight, coated with NMPA, and covered with a coverslip at 4°C for 20 min at last. After the coverslip was removed, the slides were immersed in precooled cell lysate solution away from light at 4°C overnight. When the lysates were discarded, slides were washed three times with pure water and added with appropriate helicase fluid to unwind DNA in the dark for 20 min. The slides were moved to the electrophoresis tank with buffer (300 mmol/L NaOH and 1 mmol/L EDTA, pH > 13) for 25 min and electrophoresis was conducted under 25 V, 300 mA in the dark for 20 min. Thereafter, samples were neutralized by buffer (0.4 mol/L Tris-HCl, pH 7.5) out of light for 10 min. Subsequently, slides were washed three times with pure water again. After air drying, samples were stained with suitable EB (20 *μ*g/mL) and covered with cover slips. DNA damage was evaluated with a fluorescence system of Comet Assay Software Project (a kind of comet software, Poland). The degree of DNA damage was scored by determining the percentage of DNA in the tail as follows: tail DNA% = tail DNA/(head DNA + tail DNA) × 100%.

### 2.9. Detection of Intracellular ROS Accumulation

DCFH-DA was used as a fluorescence probe to determine ROS in PC12 cells. Within the intracellular environment, DCFH-DA was deacetylated through the reaction with free radicals (H_2_O_2_) and then converted into its fluorescent product DCF. PC12 cells (2.5 × 10^5^ cells/well in a 96-well plate) were incubated at 37°C with ACR alone for 1, 3, 6, 12, and 24 h, or exposed to MT (50 *μ*mol/L) and luzindole (0.2 *μ*mol/L) at pre-24 h, simultaneously, and post-3 h with ACR (2.5 mmol/L) for 6 h. After 30 min for loading probes, intoxicated-cells were washed three times with buffer. The photos of cells cotreated with ACR and MT were taken with an inverted fluorescence microscope (scale bar: 25 *μ*m). The fluorescence intensity was examined at *λ*Ex = 488 nm and *λ*Em = 525 nm using a microplate reader.

### 2.10. Lipid Peroxidation Measurement

The MDA contents were determined by the double heating method [22], which was based on the colorimetric determination of the purple color generated by the reaction between thiobarbituric acid (TBA) and MDA. PC12 cells were incubated with ACR alone for 1, 3, 6, 12, and 24 h or treated with MT (50 *μ*mol/L) and luzindole (0.2 *μ*mol/L) at pre-24 h, simultaneously, and post-3 h with ACR (2.5 mmol/L) for 6 h. Culture supernatants in cells were collected by centrifugation, whereas fresh tissues of rats were homogenized in 0.9% normal saline to obtain supernatants with centrifugation. Supernatants were mixed with trichloroacetic acid (10%, w/v) solutions, followed by 15 min of boiling. Samples were then centrifuged at 3000 rpm for 10 min, and the supernatant was transferred to new tubes to react with TBA (0.67%, w/v) solution. After boiling for 15 min again, samples were cooled to room temperature, and the absorbance was determined at 532 nm with a microplate reader.

### 2.11. Antioxidants Determinations

Weighed brain tissues were homogenized in 0.9% normal saline and centrifuged at 2500 rpm for 10 min. The supernatants were collected for subsequent operation. The blank control, standard control, and assay group were set up. Superoxide dismutase (SOD) activity was measured based on the extent of the inhibition of amino blue tetrazolium formazan formation in the mixture of nicotinamide adenine dinucleotide, phenazine methosulfate, and nitroblue tetrazolium (NADH-PMS-NBT), according to the method described in Kakkar's study [23]. Color intensity of chromogen was determined at 560 nm. The activity unit was defined as the number of enzymes that caused 50% inhibition of NBT reduction/mg protein. GSH content in rat cerebellum was detected using 5,5-dithiobis-2-nitrobenzoic acid (DTNB) following the manufacturer's instructions. The absorbance was measured at 405 nm with a microplate reader. GSH-Px activity in rat cerebellum was measured by the rate of GSH consumption in unit time following the manufacturer's instructions. The absorbance was measured at a wavelength of 412 nm.

### 2.12. Statistical Analysis

All results were expressed as the means ± SD. All experiments were performed independently for at least six times. Statistical analysis was performed with SPSS18.0 software. Means were compared by one-way ANOVA, and groups were compared using the LSD method. Gait score was analyzed using the Mann-Whitney* U* test. Statistical significance was set at *P* < 0.05.

## 3. Results

### 3.1. MT Relieved Cytotoxicity Induced by ACR

**Figure 1:**
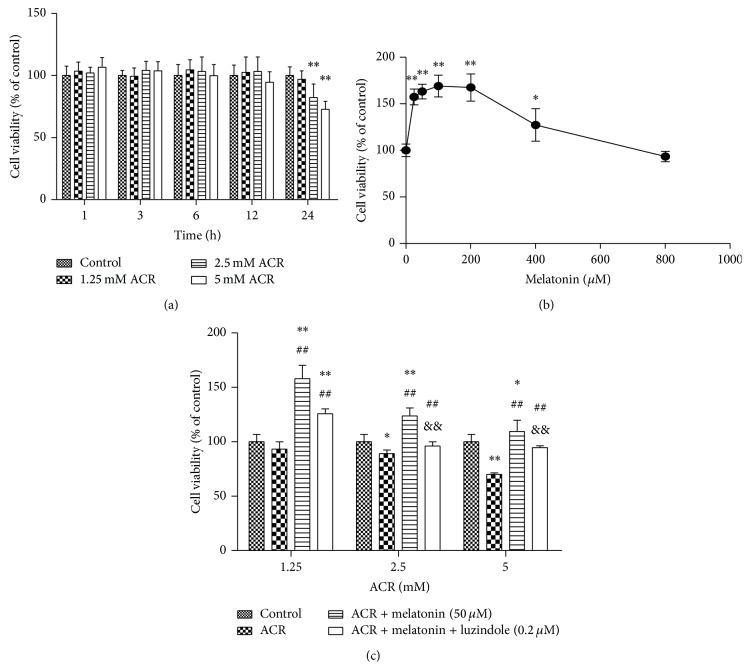
Effects of MT on cytotoxicity induced by ACR in PC12 cells. Cell viabilities were determined as relative percent of viable cells by MTT method. PC12 cells were incubated with different concentrations of ACR (1.25, 2.5, and 5 mM) for 1, 3, 6, 12, and 24 h (a) or MT (0, 25, 50, 100, 200, 400, and 800 *μ*M) for 24 h (b). Similarly, cell viabilities were analyzed in the vehicle control group, ACR alone treatment group, ACR + MT (50 *μ*M) cotreatment group, and ACR + MT + luzindole (0.2 *μ*M) cotreatment group for 24 h (c). The results are expressed as the mean ± SD (*n* = 8). ^*∗*^ *P* < 0.05, ^*∗∗*^ *P* < 0.01 versus the vehicle control group. ^#^ *P* < 0.05,  ^##^ *P* < 0.01 versus the ACR treatment group, ^&^ *P* < 0.05,  ^&&^ *P* < 0.01 versus the ACR + MT cotreatment group.

The protective effect of MT on cytotoxicity induced by ACR in PC12 cells was observed. ACR induced a statistically significant decrease in cell viability at concentrations of 2.5 and 5 mmol/L for 24 h compared with the vehicle controls (Figure 1(a)). MT at a concentration range of 25~200 *μ*mol/L exerted an increase effect on the decreased cell viability induced by ACR (Figure 1(b)). Cell viability significantly increased compared with the ACR treatment group when cells were cotreated with 50 *μ*mol/L MT and 2.5 mmol/L ACR for 24 h. Interestingly, MT receptor antagonist (0.2 *μ*mol/L luzindole) inhibited the protective effect of MT on cell viability (Figure 1(c)). Hence, MT might attenuate the cytotoxicity induced by ACR in PC12 cells potentially through the MT receptor action.

### 3.2. MT Decreased the Behavioral Toxicity in Rats and Morphological Changes in Brain Regions

**Figure 2:**
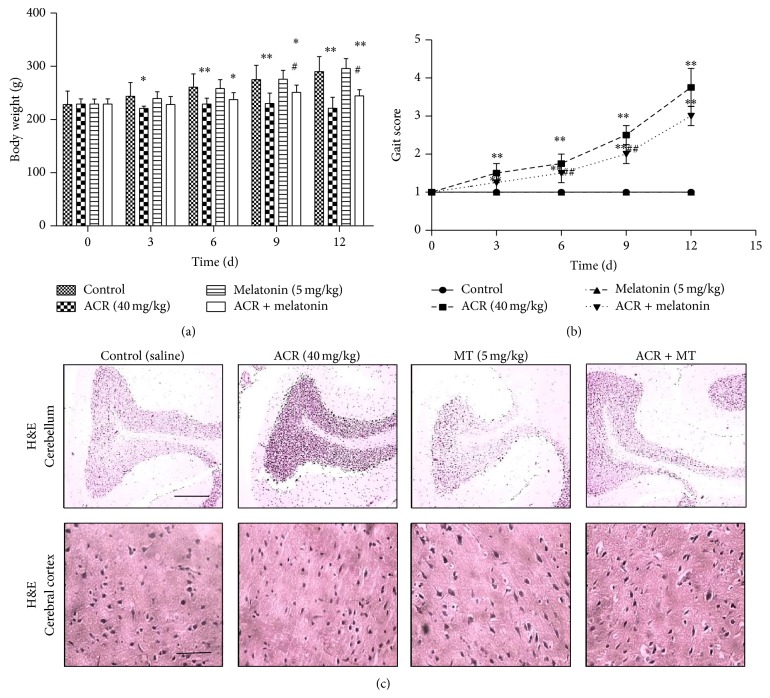
Effects of MT on behavior toxicity of rats and morphology of different brain regions. Experimental rats were divided into four groups: control (equal volume saline) group, ACR (40 mg/kg/day) treatment group, MT (5 mg/kg/day, i.p) treatment group, and ACR + MT simultaneously treatment group. Body weight (a) and gait of rats (b) were analyzed after ACR oral administration with or without MT for 0, 3, 6, 9, and 12 days. H&E staining was used to observe morphological alterations of nerve cells, and the photographs were taken under a light microscope (scale bar: 100 *μ*m) (c). The results are expressed as the mean ± SD (*n* = 8). ^*∗*^ *P* < 0.05, ^*∗∗*^ *P* < 0.01 versus the vehicle control group, ^#^ *P* < 0.05, ^##^ *P* < 0.01 versus the ACR treatment group.

To further confirm the protective effect of MT on behavioral toxicity induced by ACR, ACR poisoning rat models were established. Body weight (Figure 2(a)) and gait (Figure 2(b)) in rats were assessed, and morphological alterations in nerve cells in different brain regions were observed by H&E staining with a light microscope (Figure 2(c)). The body weight of rats at 40 mg/kg/day ACR significantly decreased since the third exposure day. When rats were cotreated with 5 mg/kg/day MT (i.p) and 40 mg/kg/day ACR, the originally reduced weight caused by ACR administration showed some improvement compared with the group treated with ACR only. Similarly, gait score in the ACR group also significantly increased since the third exposure day. However, gait score in the MT intervention group significantly decreased on days 6 and 9 of ACR treatment, compared with the ACR alone group. In addition, immunohistochemical results showed that unclear cellular gradation, concentrated cell nucleus, and decreased neurons were observed in the cortex of ACR exposure, which indicated that ACR caused abnormal lesions in the cortex of rats. However, the effect of ACR on cortex neurons was not lightened by the addition of MT. Meanwhile, the increased piriform bulging neurons and the decreased grain layer neurons were observed in Purkinje cells of cerebellum in the ACR treatment group. When MT was added, the piriform bulging neurons were reduced and karyopyknosis were weakened in Purkinje cells. Therefore, MT relieved dyskinesia, pathological changes, and karyopyknosis induced by ACR in the cerebellum of rats.

### 3.3. MT Attenuated DNA Damage Caused by ACR

**Figure 3:**
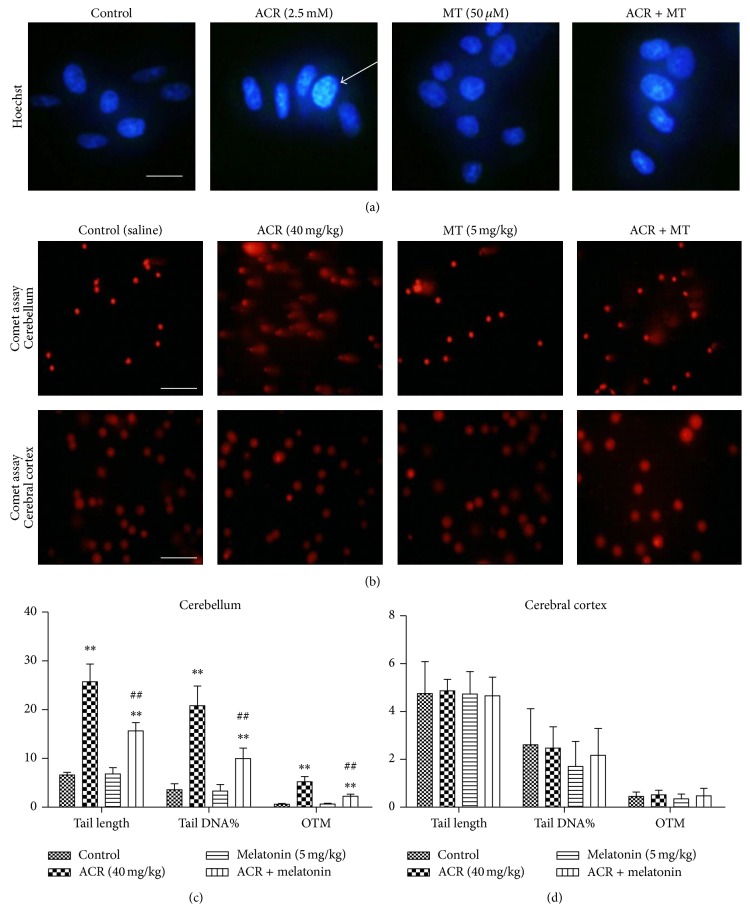
Effect of MT on ACR-induced DNA damage in PC12 cells and in rats. PC12 cells were cultured with vehicle controls, ACR (2.5 mM), MT (50 *μ*M), or ACR + MT cotreatment for 24 h. DNA condensation in cells was investigated using Hoechst 33258 nuclear staining with an inverted fluorescent microscope (scale bar: 25 *μ*m) (a). Arrows in photos refer to the positive cells. Experimental rats were divided into four groups: control (equal volume normal saline) group, ACR (40 mg·kg/day) treatment group, MT (5 mg/kg/day, i.p) treatment group, and ACR + MT simultaneously treatment group. The features of DNA damage were exhibited by a comet assay (scale bar: 50 *μ*m) (b). Tail length, tail DNA%, and olive tail-moment (OTM) were statistically analyzed to assess the degree of DNA damage in rat cerebellum (c) and cerebral cortex (d). The results are expressed as the mean ± SD (*n* = 6). ^*∗*^ *P* < 0.05,  ^*∗∗*^ *P* < 0.01 versus the vehicle control group, ^#^ *P* < 0.05,  ^##^ *P* < 0.01 versus the ACR treatment group.

DNA damage induced by ACR and the effect of MT intervention were determined by Hoechst 33258 staining in PC12 cells and comet assay in rats (Figure 3). DNA condensation and fragmentation in PC12 cells treated with 2.5 mmol/L ACR for 24 h occurred. MT at 50 *μ*mol/L prevented ACR-induced morphological alterations and inhibited these apoptotic features (Figure 3(a)). In rats, a clear and reproducible DNA fragmentation was also noted in the ACR treatment group (Figure 3(b)). The statistical results in the comet assay showed the tail length, tail DNA%, and olive tail moment (OTM) in isolated brain cells (Figures 3(c) and 3(d)). Significant increases in the tail length, tail DNA%, and OTM values in cerebellar cells with 40 mg/kg/day ACR conditioning for 12 days were observed, as compared with the vehicle controls. After MT (i.p) at 5 mg/kg/day with ACR oral administration in rats, the tail length, tail DNA%, and OTM significantly decreased compared with those in the ACR treatment group. However, no difference was found in the cerebral cortex. Thus, MT could protect ACR-induced DNA condensation and fragmentation.

### 3.4. MT Decreased ROS Accumulation and MDA Generation Produced by ACR in PC12 Cells

**Figure 4:**
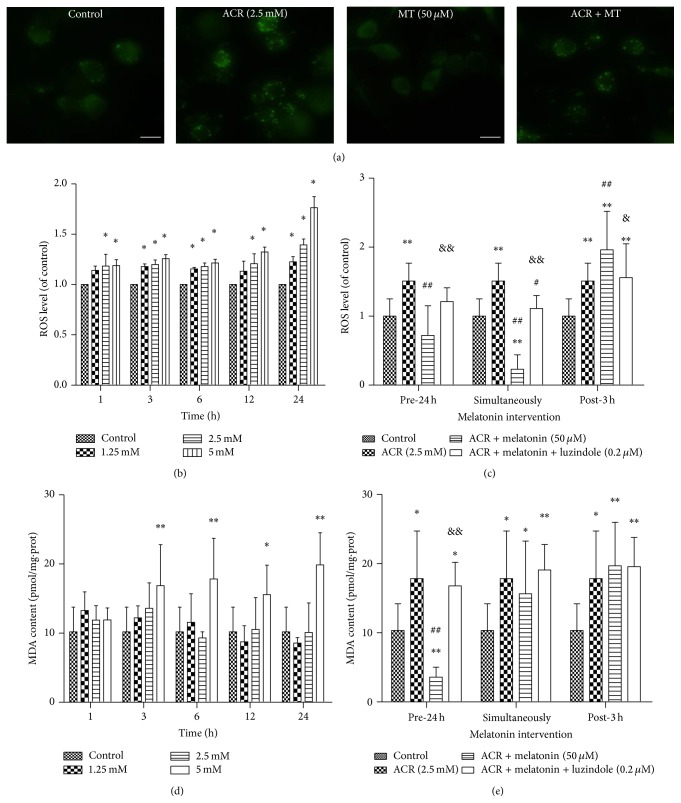
Effects of MT on ROS and MDA generations induced by ACR in PC12 cells. Intracellular ROS was evaluated by DCFH-DA detection. Fluorescent photographs were shown after exposure to ACR with or without MT cotreatment for 24 h (scale bar: 25 *μ*m) (a). In addition, ROS (b) and MDA (d) productions induced by treatment with ACR (1.25, 2.5, and 5 mM) alone for 1, 3, 6, 12, and 24 h. Moreover, MT (50 *μ*M) and luzindole (0.2 *μ*M) interventions at different times: 24 h pretreatment, simultaneously, and 3 h posttreatment with ACR (2.5 mM) for 6 h were designed ((c) and (e)). The results are expressed as the mean ± SD (*n* = 8). ^*∗*^ *P* < 0.05, ^*∗∗*^ *P* < 0.01 versus the vehicle control group. ^#^ *P* < 0.05, ^##^ *P* < 0.01 versus the ACR treatment group, ^&^ *P* < 0.05, ^&&^ *P* < 0.01 versus the ACR + MT cotreatment group.

To observe the effect of MT on oxidative stress induced by ACR, ROS accumulation and MDA generation in PC12 cells were determined. ACR exposure significantly enhanced the fluorescent intensity compared with vehicle controls in PC12 cells (Figure 4(a)). The significant increases in ROS generation were found in the 1.25 mmol/L ACR treatment group at 3 h, but in the 2.5 and 5 mmol/L ACR treatment groups at 1 h (Figure 4(b)). ROS levels increased in PC12 cells with ACR treatment in a concentration- and time-dependent manner. MT at 50 *μ*mol/L pre-24 h treatment with ACR, as well as cotreatment with ACR, protected cells from ROS accumulation induced by ACR (Figure 4(c)). MDA content at 5 mmol/L ACR treatment for 3, 6, 12, and 24 h increased significantly (Figure 4(d)). When culture cells were pretreated with MT for 24 h, ACR treatment for 24 h significantly decreased the MDA content (Figure 4(e)). Similarly, the protective effect of MT on ACR-induced ROS and MDA generation was inhibited by 0.2 *μ*mol/L luzindole pretreatment for 24 h.

### 3.5. MT Attenuated Oxidative Damage Induced by ACR in Rat Cerebellum

**Figure 5:**
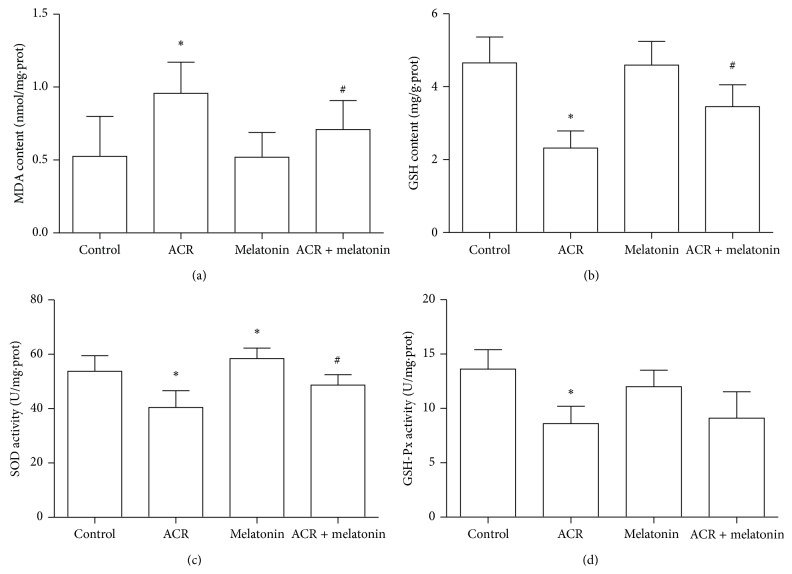
Effects of MT on MDA generation, GSH depletion, and SOD and GSH-Px activities caused by ACR in rat cerebellum. Experimental animals were divided into four groups: control (equal volume saline) group, ACR (40 mg/kg/day) treatment group, MT (5 mg/kg/day) treatment group, and ACR + MT cotreatment group. Following oral exposure for 12 days, MDA level (a), GSH content (b), SOD (c), and GSH-Px (d) activities were determined by the colorimetric method with a microplate reader. The results are expressed as the mean ± SD (*n* = 8). ^*∗*^ *P* < 0.05, ^*∗∗*^ *P* < 0.01 versus the vehicle control group, ^#^ *P* < 0.05, ^##^ *P* < 0.01 versus the ACR treatment group.

To determine the effects of MT on oxidative damage in the cerebellum of ACR (40 mg/kg b. w/day) treatment rats for 12 days, the MDA levels were measured in the cerebellum of rats. As shown in Figure 5(a), the MDA levels significantly increased in the ACR alone treatment group, compared with those in the control group. The MDA levels markedly decreased in rats orally treated with MT (5 mg/kg b. w/day) intervention compared with those in the ACR treatment group, indicating that MT inhibited MDA generation induced by ACR.

SOD activity and GSH levels were determined. As shown in Figures 5(b) and 5(c), ACR induced a significant decrease in the activities of SOD and the GSH content compared with the control group. Cerebellar SOD activity and the GSH level significantly increased in rats administered with MT compared with those in the ACR treatment group. Hence, MT exhibited the increased effects on SOD activity and GSH level in rat cerebellum. The activity of peroxide decomposition enzyme GSH-Px was also measured. As shown in Figure 5(d), GSH-Px activity significantly decreased in the ACR treatment group. However, GSH-Px activities had no significant difference between the ACR treatment group and MT (i.p) + ACR coadministration group.

## 4. Discussion

Humans are primarily exposed to ACR at low concentrations chronically through the increased intake of starchy foods. ACR has been reported to be risky for its cumulative neurotoxicity. Hence, the basic mechanisms of ACR-induced neurotoxicity on animals or humans should be illustrated. Oxidative stress is a crucial pathophysiological condition that promotes cell apoptosis and death in a broad variety of neurodegenerative disorders. Oxidative damage induced by ACR in human erythrocytes has been proposed [24]. MT, a powerful antioxidant, is likely to be protective against oxidative DNA damage induced by ACR in vitro and in vivo. However, reports on the effects of MT on ACR-induced oxidative stress are quite limited.

In the study, the differentiated PC cells induced by nerve growth factor could express neuron phenotype and characteristics and were identified to be an ideal model for studying neurotoxicity of chemicals. In cells, we focused on cell viability affected by ACR to choose the applied concentration. Cell viability in the cells treated with 2.5 mmol/L ACR for 24 h decreased significantly. ACR exposure exhibited obvious cytotoxicity on PC12 cells in a concentration- and time-dependent manner, whereas an increasing effect on cell viability was found with MT treatment for 24 h. When 50 *μ*mol/L MT and ACR were simultaneously added into the culture for 24 h, cell viability significantly increased compared with that in the ACR exposure groups. Luzindole, an effective receptor antagonism, is widely used in the study of MT mechanisms [20, 25–27]. Luzindole at 0.2 *μ*mol/L greatly inhibited the increase effect of MT on cell viability, which indicated that the receptor pathway played a vital role in the effects of MT on oxidative damage. Similarly, DNA condensation and fragmentation caused by ACR by Hoechst staining assay were observed by Hoechst staining assay, and MT co-conditioning decreased DNA fragmentation induced by ACR. MT exerted a good protective effect on ACR-induced cytotoxicity through the MT receptor pathway.

The delicate balance between the generation and catabolism of oxidants is critical for the maintenance of biological function [28]. Results demonstrated that ROS production in PC12 cells increased significantly after exposure to ACR, consistent with the results in previous studies [8, 29]. When the cells underwent MT preconditioning for 24 h or cotreatment with ACR, the decreased level of intracellular ROS was superior to that with MT posttreatment for 3 h. MDA is a decomposition product that has been used as a biomarker of lipid peroxidation for many years [30]. The MDA level was found to increase after ACR treatment alone for 3 h. This result was consistent with previous studies, where an increase in free radicals causes overproduction of MDA [11]. Therefore, MT pretreatment for 24 h prevented the development of lipid peroxidation. Interestingly, we observed that a significant increase in ROS and MDA levels induced by ACR was inhibited by receptor antagonist luzindole, indicating that luzindole exerted its channel blocking inhibitory action, and the protective effect of MT might be due to action of the MT receptors. As far as we know, MT can exert the antioxidant action mainly by membrane receptors and nuclear receptors to induce the expression of antioxidant enzymes and its ability to directly scavenge free radicals. However, in our present study, luzindole was used to test if melatonin receptor played a role in melatonin action. The results showed that blocking the receptors could not completely inhibit the protective effect of MT, surely signifying that other nonreceptor pathways might be involved in the MT activity. It was reported that MT could induce neuroprotection after focal cerebral ischemia in mice by signal pathways [21]. MT could also regulate neurodegeneration through energy metabolism and epigenetic processes in neuronal cells [31]. It was very interesting and necessary to make further researches on the mechanism of melatonin neuroprotection against ACR.

The results in cells confirmed that MT was an effective antioxidant and exerted an antagonized effect on free radicals and other oxidative products generation. To further verify the protective effect of MT on oxidative damage induced by ACR, we attempted to apply MT to observe behavioral toxicity induced by ACR to determine the used dosage. In our preliminary experiments, ACR treatment at 40 mg/kg/day for 12 days induced obvious neuropathy in rats, resulting in motor dysfunction and coordination impairment, in accordance with the previous studies [10, 32]. ACR is known to affect both the central and peripheral nervous systems in laboratory animals and humans. Hence, the gait response was considered to be the representatives of the peripheral nervous system of rats. Gait observations are a relatively sensitive parameter to evaluate neurological changes during exposure of rats to specific chemicals [33]. Consistent with our hypothesis, 40 mg/kg/day ACR subacute exposure caused weight loss of rats and motor impairment in rats. Weight loss of rats might be caused by the decreased amount of food intake or low efficiency, which was calculated by body weight gain per 100 g of food intake. Statistically significant gait changes in the presence of ACR for 3 days occurred. Results showed that MT relieved gait abnormality at 6 days and reduced the extent of weight loss at 9 days. Ataxia, an abnormality in the fine control of movement, is most commonly due to dysfunction of the cerebellum [34]. Hence, we observed pathological alterations in Purkinje cells and nuclear condensation in the cerebellum of rats, whereas no changes were observed in the cerebral cortex of rats. MT intervention restored the cerebellum lesions. This result might explain gait abnormality induced by ACR.

In previous studies in vitro, ROS levels showed a significant increase in the presence of ACR. DNA is sensitive for ROS generation induced by toxic chemicals. When ROS production and the scavenging system lose balance, excessive ROS can attack DNA macromolecules, causing oxidative DNA damage [35]. The single cell gel electrophoresis technique is a sensitive and useful for detecting DNA damage and repair in single cells [36]. Some studies showed that ACR exposure caused a significant increase in DNA migration in vitro and in vivo [37–39]. The comet assay showed that ACR administration led to apparent DNA fragmentation in the cerebellum cells, indicating that ACR had potential genotoxicity to animals after subacute exposure. MT with ACR coadministration repaired oxidative DNA damage, which revealed that MT could be used as an alternative and effective treatment as described in Ferreira's study [40]. Moreover, a significant increase in MDA level was observed in the cerebellum, suggesting the enhancement of lipid peroxidation. The results were consistent with those in PC12 cells, showing that MT exerted antioxidant action by decreasing lipid peroxidative product MDA levels in this study. When MT (i.p) with ACR cotreatment was applied, MDA products were significantly inhibited and almost recovered to the control values. Oxidative stress is usually considered as an imbalance between oxidation system and antioxidant system. ROS generation is efficiently scavenged by enzymatic antioxidant system (such as SOD and GSH-Px) and nonenzymatic antioxidants (such as GSH). SOD plays a pivotal role in the ROS defense system. Charged amino acid residues surround the center of the positively charged SOD, which creates an electric field gradient that directs O_2_^∙−^ into the center of the active enzyme through a mechanism called electrostatic guidance [41, 42]. In this study, SOD and GSH-Px activities decreased markedly in the cerebellum after 12 days of ACR treatment, leading to decrease of antioxidative function. Cotreatment with MT and ACR alleviated the decrease in SOD and GSH-Px activities. GSH, which can effectively scavenge free radicals directly and indirectly, is a major nonenzymatic antioxidant. GSH deficiency contributes to oxidative stress, which takes effect in the pathogenesis of many diseases, such as Alzheimer's disease [43]. Previous studies showed that conjugation with glutathione (GSH) was a mechanism for the detoxification of ACR [44, 45]. The results demonstrated that ACR administration caused the decrease in GSH in the cerebellum. GSH depletion might make the cerebellum more sensitive to oxidative stress. In this study, cotreatment with MT and ACR effectively prevented GSH depletion exposed to ACR for 12 days. Notably, GSH levels in the cerebellum cotreated with MT were recovered. This result was consistent with the effects of some polyphenols and antioxidants, such as curcumin, hydroxytyrosol, and tea polyphenols, on the GSH depletion in previous studies [46–50]. We infer that the protective effect of MT might be directly related to eliminating free radicals or lipid peroxide and increasing antioxidant enzymes activities through the major intracellular signal pathways. However, the specific mechanisms require further study.

## 5. Conclusions

In conclusion, our study suggested that oxidative damage induced by ACR was inhibited by MT both in vitro and in vivo. MT treatment prevented DNA fragmentation, as well as ROS and MDA overproduction induced by ACR, in PC12 cells. In rat cerebellum, MT could not only alleviate nucleus concentration and DNA damage, but also inhibit the increase in MDA levels, the decrease in SOD and GSH-Px activities, and nonenzymatic antioxidant GSH depletion caused by ACR exposure. Hence, MT is effective in preventing ACR-induced oxidative damage, which provides a good basis for beneficial applications in the chemoprevention of ACR toxicity.

## Supplementary Material

The identification of neurons was assayed by immunofluorescence in PC12 cells. The cells were fixed with 4 % paraformaldehyde for 20 min, permeabilized using 0.3 % Triton-100 in PBS for 20 min, blocked with 1 % bovine serum albumin for 30 min at room temperature, incubated with 1:200 anti-neun antibody (Neuronal marker, Abcam) at 4 oC overnight and finally probed with Alexa fluor conjugated IgG in the dark for 50 min. Microscopic images were obtained under an inverted fluorescence microscope (IX 71, Olympus, Japan), scale bar: 25 µm.

## Abbreviations

ACR: Acrylamide
MT: Melatonin
ROS: Reactive oxygen species
MDA: Malondialdehyde
SOD: Superoxide dismutase
GSH: Glutathione
GSH-Px: Glutathione peroxidase
FBS: Fetal bovine serum
DCFH-DA: 2,7-Dichlorofluorescein diacetate
MTT: 3-(4,5-Dimethylthiazol-2-yl)-2,5-diphenyltetrazolium
TBA: Thiobarbituric acid
TCA: Trichloroacetic acid
EB: Ethidium bromide
DMSO: Dimethyl sulfoxide
LMPA: Low-melting-point agarose
NMPA: Normal-melting-point agarose
DTNB: 5,5-Dithiobis-2-nitrobenzoic acid
NADH: Nicotinamide adenine dinucleotide
PMS: Phenazine methosulfate
NBT: Nitroblue tetrazolium.
