# Supplementary material for: Melatonin Attenuates Oxidative Damage Induced by Acrylamide In Vitro and In Vivo
Source: Oxid Med Cell Longev. 2015 Jun 21;2015:703709. doi: 10.1155/2015/703709 (PMC4491391; doi:10.1155/2015/703709)
Supplement: Supplementary file 1 — The identification of neurons was assayed by immunofluorescence in PC12 cells. The cells were fixed with 4 % paraformaldehyde for 20 min, permeabilized using 0.3 % Triton-100 in PBS for 20 min, blocked with 1 % bovine serum albumin for 30 min at room temperature, incubated with 1:200 anti-neun antibody (Neuronal marker, Abcam) at 4 oC overnight and finally probed with Alexa fluor conjugated IgG in the dark for 50 min. Microscopic images were obtained under an inverted fluorescence microscope (IX 71, Olympus, Japan), scale bar: 25 µm. [file 703709.f1.pdf]

**Supplementary material:**

The PC12 cells used in this study were purchased from Shanghai Institutes for Biological Sciences, Chinese Academy of Cell Resource Center (Shanghai, China). It is an approved and legal cell resource center. The cells induced by NGF have already differentiated and can express neuronal phenotype. The results indicated that the cells showed neuron characteristics.

**Immunofluorescence**

The identification of neurons was assayed by immunofluorescence in PC12 cells. The cells were fixed with 4 % paraformaldehyde for 20 min, permeabilized using 0.3 % Triton-100 in PBS for 20 min, blocked with 1 % bovine serum albumin for 30 min at room temperature, incubated with 1:200 anti-neun antibody at 4 $^{o}$C overnight and finally probed with Alexa fluor conjugated IgG in the dark for 50 min. Microscopic images were obtained under an inverted fluorescence microscope (IX 71, Olympus, Japan).

**Figure**

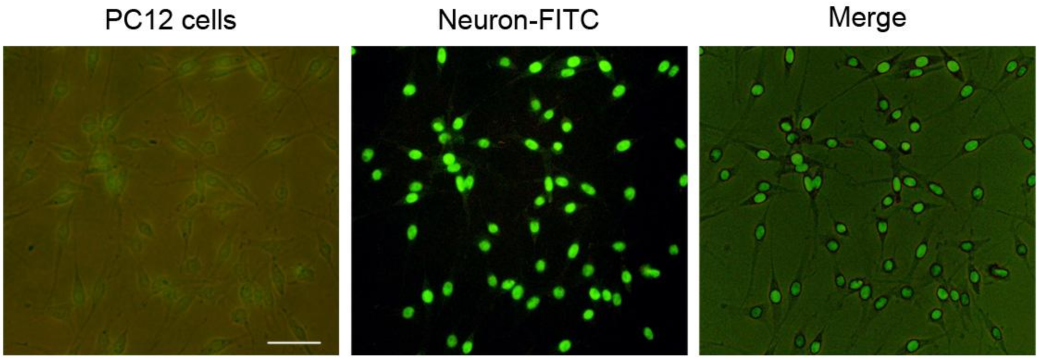


**Fig. The neuron phenotype was expressed in the differentiated PC12 cells induced by NGF**. The cells were incubated with anti-NeuN antibody (Neuronal marker, Abcam) and observed under an inverted fluorescence microscope, scale bar: 25 μm.
